# High expression of Mucin13 associates with grimmer postoperative prognosis of patients with non-metastatic clear-cell renal cell carcinoma

**DOI:** 10.18632/oncotarget.13692

**Published:** 2016-11-29

**Authors:** Zhiying Xu, Yidong Liu, Yuanfeng Yang, Jieti Wang, Guodong Zhang, Zheng Liu, Hangcheng Fu, Zewei Wang, Haiou Liu, Jiejie Xu

**Affiliations:** ^1^ Department of Biochemistry and Molecular Biology, School of Basic Medical Sciences, Fudan University, Shanghai 200032, China; ^2^ Department of Urology, Zhongshan Hospital, Fudan University, Shanghai 200032, China; ^3^ Shanghai Key Laboratory of Female Reproductive Endocrine Related Diseases, Hospital of Obstetrics and Gynecology, Fudan University, Shanghai 200011, China

**Keywords:** clear-cell renal cell carcinoma, MUC13, overall survival, recurrence-free survival, prognostic biomarker

## Abstract

**Background:**

Mucin13 (MUC13) is a transmembrane glycoprotein that is aberrantly expressed in ovarian and gastro-intestinal tumors, but its role in renal cell carcinoma remains elusive. The purpose of this study is to evaluate the prognostic value of MUC13 expression in patients with non-metastatic clear cell renal cell carcinoma (ccRCC) after surgical resection.

**Results:**

MUC13 high expression was associated with high Fuhrman grade (*p* < 0.001), high SSIGN score (*p* = 0.011), early recurrence (*p* < 0.001) and poor survival (*p* < 0.001). Multivariate Cox regression analysis identified MUC13 expression as an independent prognostic factor for RFS and OS of ccRCC patients. A nomogram integrating MUC13 expression and other independent prognosticators was established to predict RFS and OS of ccRCC patients. Optimal agreement was shown between the predictions and observations in calibration curves.

**Matrials and methods:**

This study enrolled 410 postoperative non-metastatic ccRCC patients at a single institution. Clinicopathologic variables, recurrence-free survival (RFS), and overall survival (OS) were recorded. MUC13 expression was detected by immunohistochemical staining in tumor specimens. Association of MUC13 expression with clinicopathological factors was explored. Kaplan-Meier analysis was performed to compare survival curves. Univariate and multivariate Cox regression models were used to analyze the impact of prognostic factors on RFS and OS. A prognostic nomogram was constructed based on the independent prognostic factors identified by multivariate analysis.

**Conclusions:**

MUC13 high expression is a novel independent adverse prognostic factor of clinical outcome in non-metastatic ccRCC patients after surgery.

## INTRODUCTION

In 2013, renal cell carcinoma became the seventh most common tumor, which was diagnosed in more than 350,000 people worldwide and is associated with more than 140000 deaths per year [1]. The predominant histologic subtype of RCC is clear cell renal cell carcinoma (ccRCC), which represents 75–80% of all primary kidney malignancies [2]. More than 209,000 new cases and 102,000 deaths are caused by ccRCC per year all over the world and more than one tenth patients diagnosed as ccRCC would occur fatal recurrence within 5 years after nephrectomy [3, 4]. Currently, several outcome prediction models in RCC have been proposed to evaluate the risk of disease progression of patients after nephrectomy. One widely used model based on features predictive of death from renal cell carcinoma is SSIGN (stage, size, grade, and necrosis) score, which incorporates 1997 TNM stage, tumor size, nuclear grade and histological tumor necrosis [5]. However, ccRCC patients with the similar clinical and pathological features could show a wide variation in clinical outcomes. Even though pathologic factors have been collected, their influence on the prognosis of ccRCC patients remain inconclusive [4]. These facts accentuate the urgent need for the improved predictors of RFS and OS of ccRCC patients.

Mucins are large glycoproteins providing protection and lubrication to epithelial surface of mucosal surfaces (gastrointestinal tract, respiratory tract and reproductive tract). A physical barrier created by mucins can protect epithelial cells from noxious and toxic substances. Human mucin gene family includes 19 members, which can be classified into secreted types and membrane-bound types, whereas the latter ones also participate in cell signaling [6–8]. Alterations in the expression and/or glycosylation of mucins are associated with cellular growth, differentiation, transformation, adhesion, invasion and immune surveillance. The aberrant expression of mucins may have correlation with cancer biology [6, 9, 10].

Mucin13 (MUC13) is a transmembrane mucin exhibiting abundant O- and N-glycosylation. Previous study has detected MUC13 mRNA and/or protein in normal tissues like large intestine, trachea, kidney, small intestine, gastric epithelium and esophagus. MUC13 is presented at the apical surface of cells in these tissues [11]. MUC13 consists of a signal peptide, a tandem repeat domain, three epidermal growth factor (EGF)-like domains, a SEA domain and a cytoplasmic tail domain. Tandem repeat (TR) domain, located at the N-terminus of matured MUC13, is composed of 10 TRs rich in serine and threonine residues which can be glycosylated [9]. Highly homologous with the ligand of EGFR, EGF-like domain of MUC13 is rich in cysteine residues [11]. Binding with EGFR and HER2, EGF may activate some signaling pathways resulting in alterations of cell proliferation, cell adhesion, and migration. The cytoplasmic domain of MUC13 includes several potential phosphorylation sites and a protein kinase C phosphorylation motif [11]. This indicates MUC13 may play an important role in oncogenic cellular signaling pathways. Recently, a number of studies found that MUC13 was overexpressed in many malignancies including colon cancer [12], gastric cancer [13], ovarian cancer [14], esophageal squamous cell carcinoma [15], and pancreatic cancer [16]. High expression of MUC13 also predicts an inferior outcome of patients with colorectal cancer, esophageal squamous cell carcinoma and pancreatic cancer [12, 15, 16]. Currently, the protein level and clinical significance of MUC13 expression in ccRCC remains unknown. Few studies have investigated the correlation between MUC13 expression and clinicopathologic features and outcomes of ccRCC patients.

In this study, we aimed to investigate the clinical significance of MUC13 and the association of MUC13 expression with the clinicopathological features in patients with non-metastatic ccRCC. Additionally, we integrated MUC13 expression and other prognostic variables to predict 3- and 5-year recurrence-free survival and overall survival in non-metastatic ccRCC patients.

## RESULTS

### Immunohistochemical findings and association of MUC13 expression with the clinicopathological features

**Figure 1:**
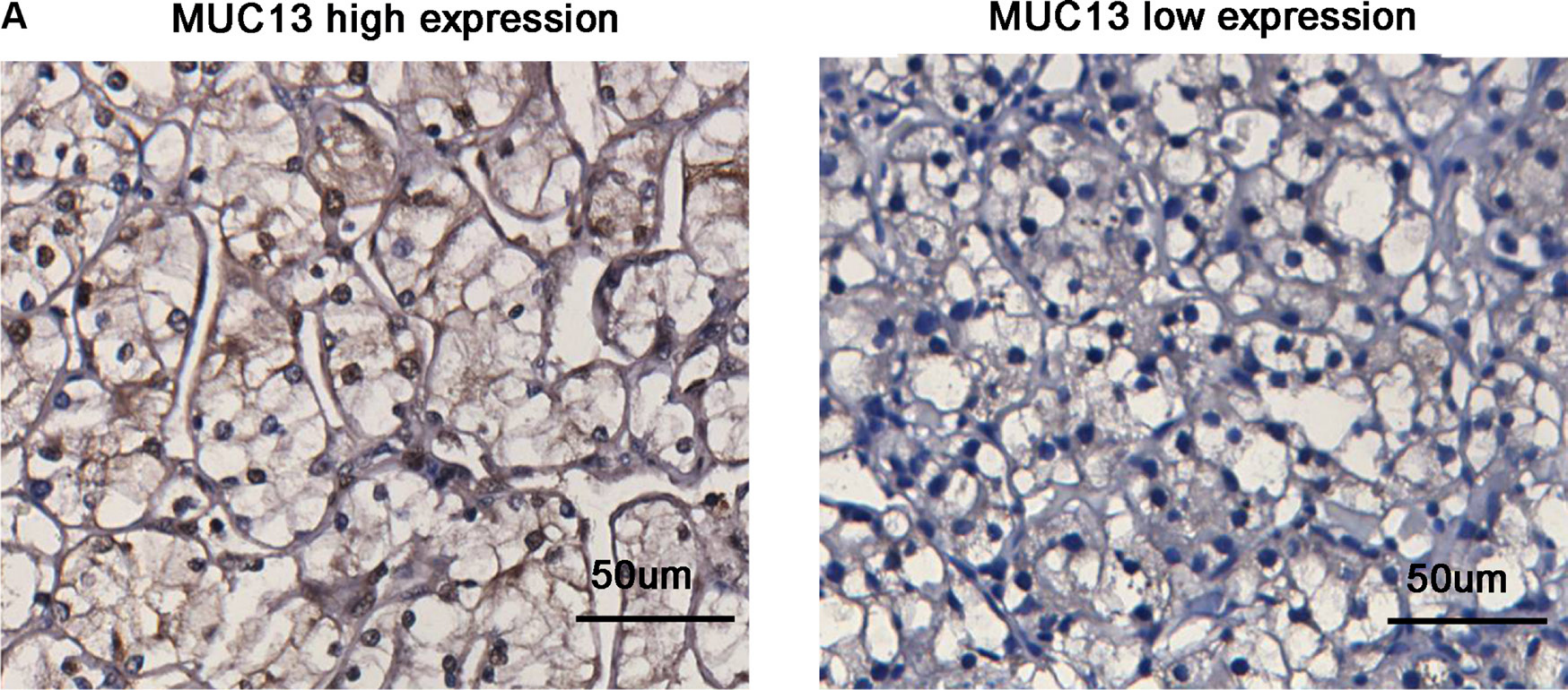
MUC13 expression in non-metastatic clear-cell renal cell carcinoma (ccRCC) tissues (**A**) Representative photographs of immunohistochemical (IHC) staining in ccRCC tissues with differential expression level of MUC13. Scale bar = 50 μm.

MUC13 expression levels was identified by immunohistochemical staining in 410 non-metastatic ccRCC specimens. We observed that MUC13 was not only expressed on the apical surface but also located in the cytoplasm and in the nucleus of tumor cells (Figure 1A). The immunohistochemical score (H-score) of immunostaining of tumor tissues differed among each specimen. The average measured H-score of the staining in neoplasm was 95.5 (range 0.6–280.6). The cut-off point for the classification of high/low expression subgroups was 92.0. 198 (48.3%) patients were classified into MUC13 high expression subgroup while the MUC13 low expression subgroup had 212 (51.7%) patients.

As summarized in Table 1, the mean age in the entire group was 55.21 ± 12.24 years old (range 21–86) and the average tumor size was 4.14 ± 2.26 cm (range 0.2–15). 70.5% of patients were male. Table 1 also presents the correlation between MUC13 expression and clinical pathological characteristics. Higher MUC13 expression showed positive association with elevated Fuhrman grade (*p* < 0.001) and higher SSIGN score (*p* = 0.011). We failed to observe the association between other clinical pathological characteristics and MUC13 expression.

**Table 1:** Correlation between MUC13 expression and patient characteristics

Characteristic	Patients (*n* = 410)		MUC13 expression		*P*^a^
	Number	%	Low (*n*= 212)	High (*n*= 198)	
Age at surgery, years^b^					0.152
Mean ± SD	55.21 ± 12.24		53.16 ± 12.64	57.40 ± 11.43	
Gender					0.675
Female	121	29.5	65	56	
Male	289	70.5	147	142	
Tumor size, cm^b^					0.109
Mean ± SD	4.14 ± 2.26		3.02 ± 2.13	4.39 ± 2.38	
pT-stage					0.094
1	290	70.7	156	134	
2	24	5.9	15	9	
3	96	23.4	41	55	
Fuhrman grade					**< 0.001**
1	74	18.0	57	17	
2	197	48.1	101	96	
3	93	22.7	43	50	
4	46	11.2	11	35	
LVI					0.772
Absent	306	74.6	160	146	
Present	104	25.4	52	52	
Necrosis					0.930
Absent	331	80.7	172	159	
Present	79	19.3	40	39	
Sarcomatoid					0.649
Absent	402	98.0	209	193	
Present	8	2.0	3	5	
Rahbdoid					0.157
Absent	394	96.1	207	187	
Present	16	3.9	5	11	
ECOG-PS					0.169
0	343	83.7	183	160	
≥ 1	67	16.3	29	38	
SSIGN					**0.011**
1	305	74.4	169	136	
2	97	23.6	39	58	
3	8	2.0	2	6	

MUC13 = mucin13; SD = standard deviation;

LVI = Lymphovascular invasion; ECOG-PS = Eastern Cooperative Oncology Group performance status;

SSIGN = stage, size, grade and necrosis.

^a^*P* < 0.05 is considered statistically significant.

^b^The results of continuous variables are presented as mean ± SD (standard deviation).

### Correlations between MUC13 expression and prognosis of ccRCC patients

**Figure 2:**
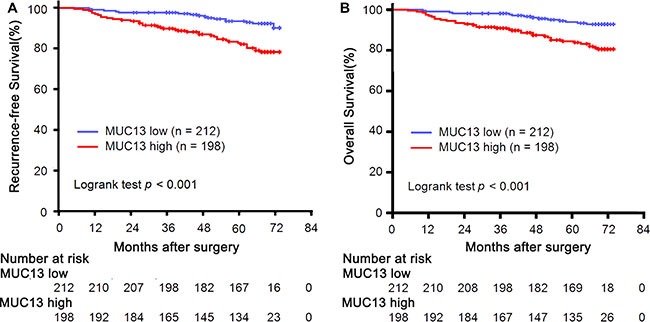
Analysis of RFS and OS of patients with non-metastatic ccRCC according to MUC13 expression in all patients (**A**) Kaplan-Meier analysis of RFS (*n* = 410, *p* < 0.001). (**B**) Kaplan-Meier analysis of OS (*n* = 410, *p* < 0.001). *P-value* was calculated by log-rank test.

At last follow up, median follow-up for patients was 70 months (range 42–74). A mean duration of recurrence-free survival (RFS) was 62 months (range 5–74) and overall survival (OS) was 62 months (range 5–74). Kaplan-Meier analyses log-rank test illustrated that high MUC13 expression could predict earlier recurrence and worse overall survival (*p* < 0.001, *p* < 0.001, respectively) (Figure 2A, 2B).

**Figure 3:**
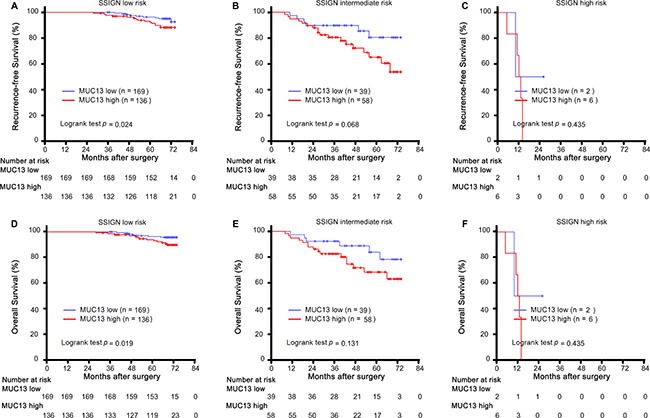
Analysis of RFS and OS according to MUC13 expression in each SSIGN risk group (**A–C**) Kaplan-Meier analysis of RFS according to MUC13 expression in (A) SSIGN low risk, (B) SSIGN intermediate risk, (C) SSIGN high risk patients. (**D–F**) Kaplan-Meier analysis of OS according to MUC13 expression in (D) SSIGN low risk, (E) SSIGN intermediate risk, (F) SSIGN high risk patients. *P-value* was calculated by log-rank test.

Furthermore, in order to estimate whether patients can be stratified by MUC13 expression with SSIGN score stratum. Patients were stratified into three risk subgroups: low risk (SSIGN score: 1–2; *n* = 305, 74.4%), intermediate risk (SSIGN score: 3–4; *n* = 97, 23.7%) and high risk (SSIGN score: 5–6; *n* = 8, 2.0%). When the analysis was restricted to low risk group, patients could be significantly stratified with MUC13 expression. High MUC13 expression correlated with decreased recurrence-free survival and reduced overall survival (*p* = 0.024, *p* = 0.019, respectively) (Figure 3A, 3D). However, in intermediate risk group and high risk group, the difference didn't remain significant in recurrence-free survival or overall survival (*p* = 0.068, *p* = 0.435, *p* = 0.131, *p* = 0.435, respectively) (Figure 3B, 3C, 3E, 3F).

### High MUC13 expression is an independent predictor of poor prognosis in patients with ccRCC

Univariate analyses were performed for RFS and OS to estimate the clinical significance of MUC13 expression on postoperative survival in the study group. According to the Supplementary Table S1, we observed that high MUC13 expression significantly correlated with reduced RFS and worse OS (HR, 2.952; 95% CI, 1.588 to 5.488, *p* < 0.001 and HR, 2.890; 95% CI, 1.614 to 5.172, *p* < 0.001, respectively). Additionally, tumor size, pT stage, Fuhrman grade, LVI, necrosis, sarcomatoid, rahbdoid and ECOG-PS also significantly influenced RFS and OS of patients with ccRCC.

In addition, to obtain the robustness value of MUC13 expression, multivariate Cox regression analyses were performed to derive risk evaluation related to OS and RFS with cilnicopathologic parameters derived from univariate analyses Table 2. PT stage, Fuhrman grade, LVI and necrosis, high MUC13 expression (HR, 2.082; 95% CI, 1.115 to 3.889, *p* = 0.021) were independent predictors of RFS. Together with pT stage, Fuhrman grade, LVI, necrosis and rahbdoid, high MUC13 expression (HR, 2.287; 95% CI, 1.169 to 4.477, *p* = 0.016) also remained an independent prognostic factor for OS. In total, our study illustrated that MUC13 expression might be an independent indicator to predict recurrence-free survival and overall survival of non-metastatic ccRCC patients. The C-index of the SSIGN was 0.7440 for OS and 0.7336 for RFS, and improved to 0.7933 for OS (*p* = 0.009) and 0.7836 for RFS (*p* = 0.006) when MUC13 expression was added.

**Table 2:** Multivariate cox regression analysis of recurrence-free survival and overall survival

Characteristic	Recurrence-free survival (*n* = 410, event = 55)		Overall survival (*n* = 410, event = 49)	
	Hazard Ratio (95% CI)	*P*^a^	Hazard Ratio (95% CI)	*P*^a^
Tumor size, cm	1.368 (1.192–1.571)	<**0.001**	1.327 (1.155–1.525)	<**0.001**
pT-stage		**0.006**		<**0.001**
1	Reference		Reference	
2	1.528 (0.434–5.378)	0.509	3.136 (0.933–10.542)	0.065
3	3.177 (1.547–6.526)	**0.002**	4.826 (2.272–10.251)	<**0.001**
Fuhrman grade		**0.004**		**0.029**
1	Reference		Reference	
2	1.232 (0.351–4.323)	0.745	1.028 (0.289–3.657)	0.966
3	2.366 (0.649–8.618)	0.192	1.593 (0.416–6.098)	0.497
4	4.959 (1.350–18.213)	**0.016**	3.600 (0.950–13.642)	0.060
LVI		<**0.001**		<**0.001**
Absent	Reference		Reference	
Present	2.973 (1.591–5.556)		3.355 (1.712–6.574)	
Necrosis		**0.002**		**0.009**
Absent	Reference		Reference	
Present	2.679 (1.422–5.049)		2.515 (1.258–5.028)	
Sarcomatoid		0.184		0.255
Absent	Reference		Reference	
Present	2.394 (0.659–8.692)		2.150 (0.576–8.030)	
Rahbdoid		0.378		**0.013**
Absent	Reference		Reference	
Present	1.514 (0.602–3.809)		2.990 (1.264–7.073)	
ECOG-PS		0.248		0.196
0	Reference		Reference	
≥ 1	1.479 (0.761–2.875)		1.605 (0.784–3.286)	
MUC13		**0.021**		**0.016**
Low	Reference		Reference	
High	2.082 (1.115–3.889)		2.287 (1.169–4.477)	

MUC13 = mucin13; CI = confidence interval;

LVI = Lymphovascular invasion; ECOG-PS = Eastern Cooperative Oncology Group performance status.

^a^*P*< 0.05 is considered statistically significant.

### Construction and validation of prognostic nomogram for RFS and OS

**Figure 4:**
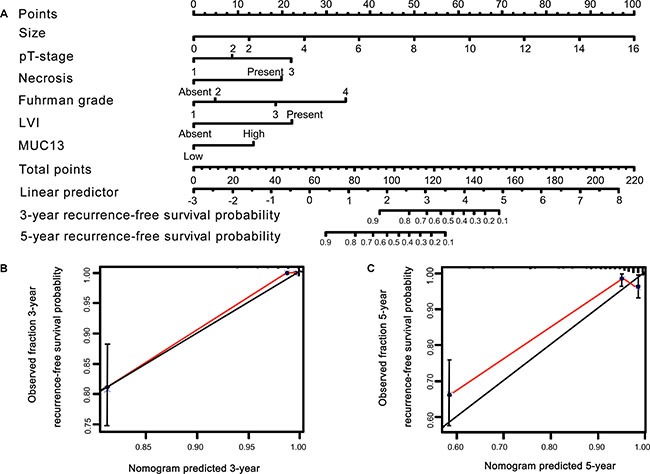
Nomogram and calibration plot for RFS prediction in patients with non-metastatic ccRCC (**A**) Five independent prognostic factors were entered into the nomogram to predict 3- and 5-year recurrence-free survival. The calibration plots for predicting RFS at (**B**) 3 years, (**C**) 5 years.

**Figure 5:**
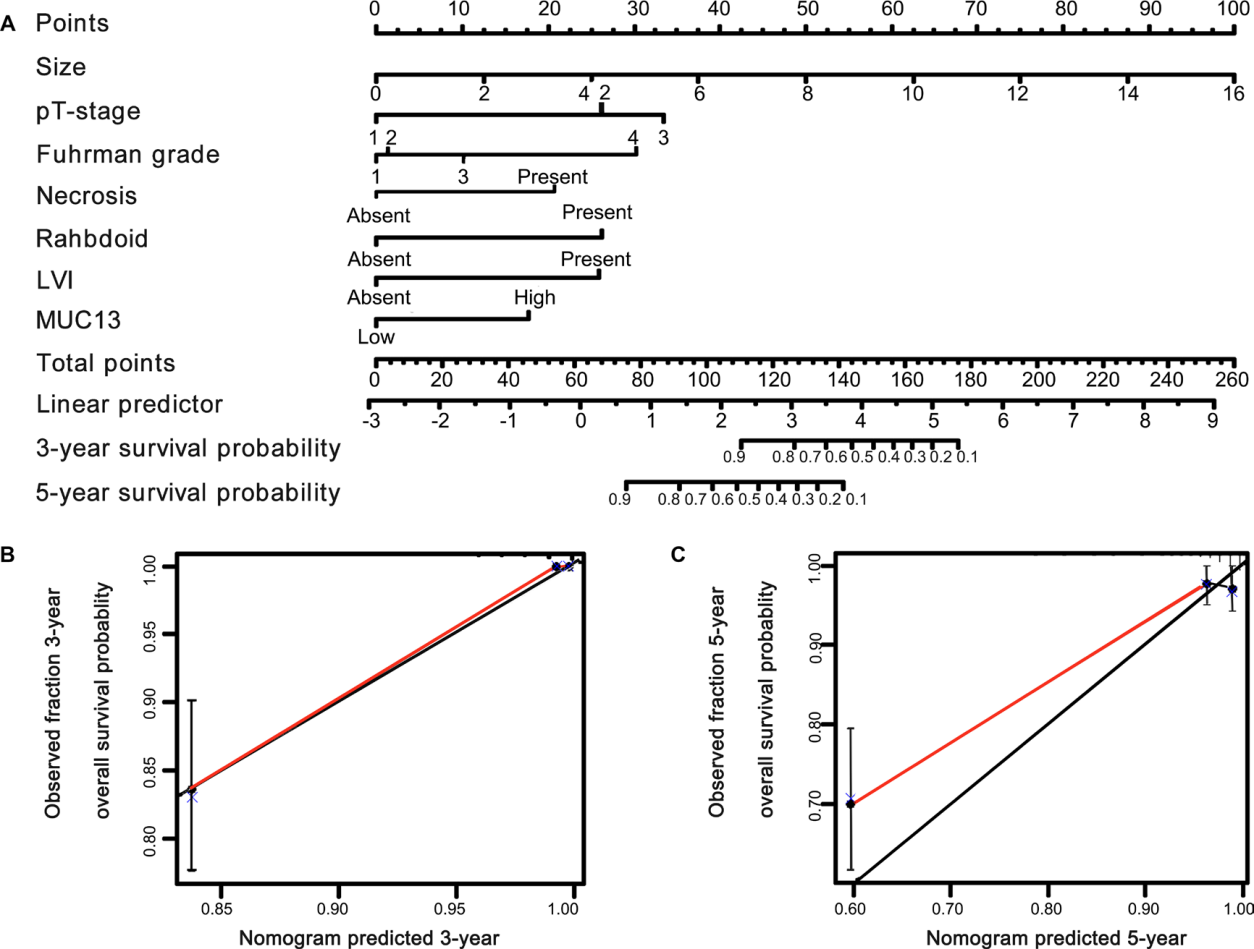
Nomogram and calibration plot for OS prediction in patients with non-metastatic ccRCC (**A**) Six independent prognostic factors were entered into the nomogram to predict 3- and 5-year overall survival. The calibration plots for predicting OS at (**B**) 3 years, (**C**) 5 years.

Significant prognostic factors were concluded from multivariate Cox regression analyses of OS and RFS to establish nomogram (Figures 4A, 5A). The calibration plot for the nomogram presented an optimal agreement between the predicted and actual observation for the RFS and OS at 3-yr and 5-yr (Figures 4B, 4C, 5B, 5C). We also preformed Harrell's c-index to evaluate the prognostic accuracy for the nomogram. The Harrell's c-index for the nomogram model to predict 3-year and 5-year overall survival were 0.9668 and 0.8697, respectively. And the Harrell's c-index for the nomogram model to predict 3-year and 5-year recurrence-free survival were 0.9743 and 0.8701, respectively.

## DISCUSSION

As far as we know, this study was the first investigation illustrating the correlation between high MUC13 expression and unfavorable prognosis of postoperative non-metastatic ccRCC patients. We found that incorporation of MUC13 expression into SSIGN model could improve the stratification of patients and provide more prognostic information. Our study demonstrated that MUC13 expression was positively correlated to Fuhrman grade, which implies that MUC13 might be associated with differentiation of ccRCC. In addition, MUC13 expression was evaluated as an independent prognostic factor and could be incorporated into a nomogram with other established pathologic factors. The study indicated that MUC13 might promote the tumorigenesis and development of ccRCC.

MUC13 is a high-molecular-weight glycoproteins of mucin family. The existence of TR domain-the hallmark feature of this family, indicates that the extracellular part of the protein may be capable of blocking cell–cell adhesion and cell adhesion to the ECR through protruding more than 200 to 2,000 nm above the cancer cell surface. Hence, the overexpression of mucins may be involved in the exfoliation, dissemination, and invasion of cancer cells [6]. Consistent with our results, a growing body of literatures found that overexpression and aberrant localization of MUC13 was observed in other cancers [12–16]. One of these studies illustrated that exogenous MUC13 expression correlated with remarkable reduction in cell-cell adhesion and significantly (*p* < 0.05) increases cell motility, proliferation, and tumorigenesis in a xenograft mouse model system with ovarian cancer. And the cellular characteristics were associated with up-regulation of HER-2, p21-activated kinase 1, and p38 protein expression [14]. This phenomenon implies that the combination of HER-2 and EDF-like domain may be implicated in the modulations of cellular characteristic and MUC13 may play a significant role in the development of cancer. But the correlation between MUC13 and ccRCC cellular characteristic still needs more investigation.

Mucins have been demonstrated as potential tumor markers and therapeutic targets [17–19], because of their potential for altered glycosylation patterns and abundant expression in cancer. Our previous studies found that high expression of mucin3A or mucin7 can predict poor prognosis of ccRCC patients while decreased expression of mucin4 or mucin18 is associated with poor outcome of ccRCC patients [20–23]. Increased expression of mucin1 was observed in ccRCC and may play a role in renal cancer progression [24]. The biological role of mucin1 in renal cancer has been studied for a long time and was found as an actor in epithelial-mesenchymal transition which may enable invasion and initiate metastasis of renal carcinoma [25]. Under hypoxia, binding of HIF-1 alpha and mucin1 promoter leads to overexpression of mucin1 which can promote the invasive and migration properties of ccRCC [26]. And mucin1-C nuclear location drives invasiveness of renal cancer cell by a sheddase/gamma secretase dependent mechanism [27]. Previous study demonstrated that blockage of MUC13 decreased sensitivity of esophageal squamous cell carcinoma cell lines to paclitaxel [28]. MUC13 can promote nuclear factor-κB (NF-κB) activation to prolong colorectal cancer cell survival. High expression of cytoplasmic MUC13 and NF-κB is associated with progression and metastases of colorectal cancer. Silencing MUC13 can improve the sensitivity of colorectal cancer cells to chemotherapy. These indicate that MUC13 may become a novel prognostic biomarker and predict for chemosensitivity of cancer [29]. Extracellular luminal staining data of a previous study implied that it was possible for MUC13 to release into the secretions and/or blood after shedding from the ovarian cancer cells [14]. Therefore, MUC13 serum and urine immunoassay might be developed as a screening method or diagnostic marker for cancer. High expression of MUC13 is regard as an independent prognostic indicator of early-staged gastric cancer [30]. In the current study, our initial work revealed that only the patients in SSIGN low risk subgroups could be significantly stratified by MUC13 expression. Therefore MUC13 may provide more details to clinicians for changing the frequency of follow-up and improving therapeutic approaches for SSIGN low risk subgroups ccRCC patients. Recently, a study found that high expression of MUC13 was associated with deceased expression of tumor suppressor, p53, and also the activation or expression of crucial oncogenes, HER2, PAK1, ERK, Akt, and S100A4 [16]. Another study demonstrated that miR-145 can suppress tumor growth of pancreatic cancer through its inhibitory effects on MUC13 [31]. These implied that MUC13 might become an effective therapeutic target for cancer.

Although we have demonstrated the clinical significance of MUC13 expression in ccRCC, it is undeniable that several limitations in this study warrant further discussion. Firstly, we have to acknowledge the statistical limitations of the study due to small number of patients, especially for whom with advanced disease. Secondly, an independent external cohort is required to confirm our research findings. Finally, further researches are required to confirm the mechanisms of MUC13 in the tumorigenesis of ccRCC and its potential to become a drug target.

In conclusion, our observations revealed that high MUC13 expression can independently predicts unfavorable postoperative RFS and OS for ccRCC patients. A prognostic nomogram integrating MUC13 expression and other pathologic factors was constructed to predict the RFS and OS of ccRCC patients. Furthermore, MUC13 might become an immunotherapeutic target for ccRCC. An intensive focus is required to confirm the biological mechanism of MUC13 involved in ccRCC progression and management, which may reveal promising therapeutic strategies for ccRCC treatment.

## MATERIALS AND METHODS

### Patients

410 patients diagnosed with non-metastatic clear-cell RCC histopathologically were enrolled in the study. The patients underwent partial or radical nephrectomy between Jan 7, 2008 and Dec 23, 2009 at Zhongshan Hospital, Fudan University, Shanghai, China. Patients who have confirmed histopathology diagnosis after nephrectomy, complete available follow-up data and suffered from no comorbidities, received no adjuvant post-operative anticancer therapy are included in this study. And patients were excluded based on the following criteria: (a) incomplete follow-up data; (b) bilateral disease and familial RCC; (c) preoperative neoadjuvant and/or postoperative adjuvant therapy; (d) death within the first month after operation. Corresponding Formalin Fixed Paraffin Embedded specimen of tumor mass (≥ 1 cm^3^) were achieved and kept in the archives. The specimen were classified into different histological subtypes according to 2014 EAU guidelines [32]. No positive margins were reported in the pathological reports of patients.

All clinicopathologic and baseline demographic characteristics factors and follow-up outcomes were collected in the database. The pT-stage was carried out based on TNM classification of AJCC (American Joint Committee on Cancer) in 2010 [33]. Fuhrman grade, LVI (lymphovascular invasion), necrosis, sarcomatoid features and rahbdoid features were recorded according the 2012 ISUP (International Society of Urological Pathology) consensus [4]. Eastern ECOG-PS (Eastern Cooperative Oncology Group - performance status) were estimated as previously reported [34]. SSIGN scores were evaluated according to original scoring algorithm [35]. Overall survival (OS) was defined as the time from surgery to the death or the last follow-up. Recurrence-free survival (RFS) was calculated from the date of surgery to the first RCC recurrence. Data were censored if the patients kept alive at the end of the follow-up. The study had achieved the informed consent of all patients and the approval from the institutional ethical review boards of hospital.

### Tissue microarray construction and immunohistochemistry

Tissue microarrays (TMA) were constructed as previously described [20]. Duplicate 1.0-mm tissue cores from two different areas were used to construct the TMA. Primary Anti-MUC13 antibody (1:500; Abam, Cambridge, MA, USA) are applied for the IHC (immunohistochemical) staining. A fully automated microscopy system (Leica DM6000 B, Leica Microsystems GmbH, Mannheim, Germany) was used to scan immunohistochemistry sections. We measured the density of positive staining by Leica Ariol 4.0 software. These mi-quantitative H-score, ranged from 0 to 300, was derived from the multiplication of the staining intensities (0: negative, 1: weak staining, 2: moderate staining, 3: strong staining) and the distributions (0–100%) For each specimen, the mean score of duplicates was adopted for statistical analyses. The H-score was evaluated by two independent pathologists without the knowledge of clinicopathological information. The agreement between the two twp was excellent, which was evaluated by kappa value (0.85).

### Statistical analysis

Analyses were performed with X-tile software version 3.6.1 (Yale University, New Haven, CT), MedCalc Software version 12.7.0 (MedCalc, Mariakerke, Belgium), SPSS version 21.0 (IBM, Armonk, NY), Stata SE version 14 (Stata, College Station, TX) and R software packages version 3.2.3 (The R Foundation for Statistical Computing,http://www.r-project.org/). A minimum *P-value* approach calculated by X-tile software was applied to determine the cut-off point. Correlations between MUC13 expression and clinicopathologic variables were analyzed by Student's *t* test, Pearsonχ2 test and Fisher's exact test, as appropriate. Survival curves were determined by Kaplan-Meier analysis and compared by Log-rank test. Univariate and multivariate analyses were performed by the stepwise Cox proportional hazard regression model. Harrell's concordance index (C-index) was used to evaluate the predictive accuracy of parameters and prognostic models. Nomogram was constructed as the prognostic model whose accuracy was evaluated by the Calibration plot. All data tests were two-sided and differences were considered significant when *p value* was under 0.05.

## SUPPLEMENTARY MATERIALS FIGURES AND TABLES





## Abbreviations

MUC13: Mucin13
ccRCC: Clear-cell renal carcinoma
RFS: Recurrence-free survival
OS: Overall survival
SSIGN: Mayo clinic stage, size, grade, and necrosis score
RCC: Renal cell carcinoma
EGF: Epidermal growth factor
TR domain: Tandem repeat domain
HER2: Human epidermal growth factor receptor 2
LVI: Lymphovascular invasion
ECOG PS: Eastern cooperative oncology group performance status
C index: Harrell's concordance index
H-score: immunohistochemical score
HR: Hazard ratio
