# Supplementary material for: High expression of Mucin13 associates with grimmer postoperative prognosis of patients with non-metastatic clear-cell renal cell carcinoma
Source: Oncotarget. 2016 Nov 29;8(5):7548–58. doi: 10.18632/oncotarget.13692 (PMC5352342; doi:10.18632/oncotarget.13692)
Supplement: Supplementary file 1 [file oncotarget-08-7548-s001.pdf]

High expression of Mucin13 associates with grimmer postoperative prognosis of patients with non-metastatic clear-cell renal cell carcinoma

Supplementary Materials

Supplementary Table S1: Univariate cox regression analysis of recurrence-free survival and overall survival

Characteristic	Recurrence-free survival		Overall survival	
	Hazard Ratio (95% CI)	P^a	Hazard Ratio (95% CI)	P^a
Tumor size, cm	1.564 (1.416–1.729)	**< 0.001**	1.551 (1.408–1.709)	**< 0.001**
pT-stage		**< 0.001**		**< 0.001**
1	Reference		Reference	
2	5.120 (1.918–13.666)		3.241 (1.128–9.311)	
3	4.063 (2.237–7.380)		3.163 (1.801–5.553)	
Fuhrman grade		**< 0.001**		**< 0.001**
1	Reference		Reference	
2	1.809 (0.520–6.295)		1.953 (0.565–6.745)	
3	4.665 (1.327–16.398)		5.507 (1.592–19.054)	
4	18.296 (5.387–62.139)		22.085 (6.579–74.134)	
LVI		**< 0.001**		**< 0.001**
Absent	Reference		Reference	
Present	5.160 (2.923–9.110)		4.808 (2.819–8.199)	
Necrosis		**< 0.001**		**< 0.001**
Absent	Reference		Reference	
Present	4.677 (2.657–8.233)		4.928 (2.891–8.402)	
Sarcomatoid		**< 0.001**		**< 0.001**
Absent	Reference		Reference	
Present	17.030 (5.679–51.073)		16.455 (5.511–49.128)	
Rahbdoid		**< 0.001**		**< 0.001**
Absent	Reference		Reference	
Present	10.260 (4.892–21.520)		6.791 (3.028–15.232)	
ECOG-PS		**< 0.001**		**< 0.001**
0	Reference		Reference	
≥ 1	4.802 (2.658–8.675)		4.511 (2.561–7.948)	
MUC13		**< 0.001**		**< 0.001**
Low	Reference		Reference	
High	2.952 (1.588–5.488)		2.890 (1.614–5.172)	

Abbreviations: MUC13 = mucin13; CI = confidence interval;
LVI = Lymphovascular invasion; ECOG PS = Eastern Cooperative Oncology Group performance status.
[a] $P < 0.05$ is considered statistically significant.
