# Supplementary material for: High expression of Mucin13 associates with grimmer postoperative prognosis of patients with non-metastatic clear-cell renal cell carcinoma
Source: Oncotarget. 2016 Nov 29;8(5):7548–58. doi: 10.18632/oncotarget.13692 (PMC5352342; doi:10.18632/oncotarget.13692)
Supplement: Supplementary file 2 [file oncotarget-08-7548-s002.docx]

| **Supplementary Table S1: Univariate cox regression analysis of recurrence-free survival and overall survival** | | | | |
| --- | --- | --- | --- | --- |
| **Characteristic** | **Recurrence-free survival** | | **Overall survival** | |
|  | **Hazard Ratio (95% CI)** | ***P*^a^** | **Hazard Ratio (95% CI)** | ***P*^a^** |
| Tumor size, cm | 1.564 (1.416–1.729) | **<** **0.001** | 1.551 (1.408–1.709) | **< 0.001** |
| pT-stage   1   2   3 | Reference  5.120 (1.918–13.666)  4.063 (2.237–7.380) | **< 0.001** | Reference  3.241 (1.128–9.311)  3.163 (1.801–5.553) | **< 0.001** |
| Fuhrman grade   1   2   3   4 | Reference  1.809 (0.520–6.295)  4.665 (1.327–16.398)  18.296 (5.387–62.139) | **< 0.001** | Reference  1.953 (0.565–6.745)  5.507 (1.592–19.054)  22.085 (6.579–74.134) | **< 0.001** |
| LVI   Absent   Present | Reference  5.160 (2.923–9.110) | **< 0.001** | Reference  4.808 (2.819–8.199) | **< 0.001** |
| Necrosis   Absent   Present | Reference  4.677 (2.657–8.233) | **< 0.001** | Reference  4.928 (2.891–8.402) | **< 0.001** |
| Sarcomatoid   Absent   Present | Reference  17.030 (5.679–51.073) | **< 0.001** | Reference  16.455 (5.511–49.128) | **< 0.001** |
| Rahbdoid   Absent   Present | Reference  10.260 (4.892–21.520) | **< 0.001** | Reference  6.791 (3.028–15.232) | **< 0.001** |
| ECOG-PS   0   ≥ 1 | Reference  4.802 (2.658–8.675) | **< 0.001** | Reference  4.511 (2.561–7.948) | **< 0.001** |
| MUC13   Low   High | Reference  2.952 (1.588–5.488) | **< 0.001** | Reference  2.890 (1.614–5.172) | **< 0.001** |
| Abbreviations: MUC13 = mucin13; CI = confidence interval;  LVI = Lymphovascular invasion; ECOG PS = Eastern Cooperative Oncology Group performance status.  ^a^*P* < 0.05 is considered statistically significant. | | | | |
